# Covalently Bound MOF/COF Aerogels as Robust Catalytic Filters for Rapid Nerve Agent Decomposition

**DOI:** 10.1021/acsami.4c19759

**Published:** 2025-03-03

**Authors:** Martin Sahul’, Youven Benseghir, Tanja Eder, Flora Schöfbeck, Lingcong Ge, Dániel Hetey, Michael R. Reithofer, Jia Min Chin

**Affiliations:** † Institute of Functional Materials and Catalysis, Faculty of Chemistry, University of Vienna, Währinger Str. 42, 1090 Vienna, Austria; ‡ Vienna Doctoral School in Chemistry (DoSChem), University of Vienna, Währinger Str. 42, 1090 Vienna, Austria; § Institute of Inorganic Chemistry, Faculty of Chemistry, University of Vienna, Währinger Str. 42, 1090 Vienna, Austria

**Keywords:** metal−organic frameworks, covalent organic frameworks, aerogels, organophosphate degradation, porosity, catalysis

## Abstract

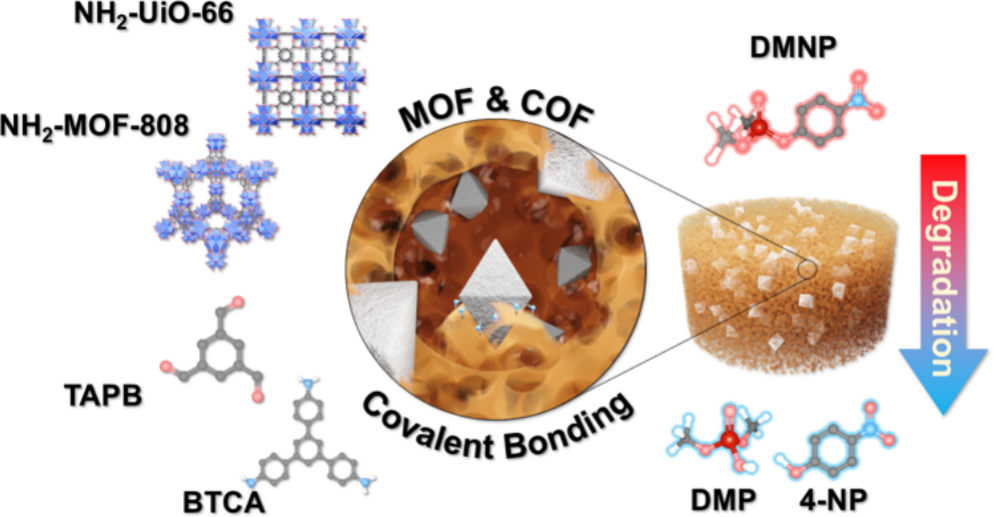

Metal–organic frameworks (MOFs) show promising results in various fields, such as gas separation and catalysis, but they face limitations due to problems associated with their low processability. This study addresses these challenges by utilizing postsynthetic modification (PSM) of NH_2_–UiO-66 and NH_2_-MOF-808 with 1,3,5-benzene tricarbaldehyde (BTCA) to form hybrid aerogels consisting of MOF-loaded covalent organic framework (COF). BTCA-modified MOF nanoparticles via imine bond formation were confirmed by ^1^H NMR, FTIR, and solid-state ^13^C NMR spectroscopies. MOF/COF composites were analyzed via TGA, PXRD, BET, and solid-state ^13^C NMR, showing retained crystallinity and increased porosity in comparison to the sum of the individual components. Moreover, improved aerogel mechanical properties and increased MOF loading of up to 75 wt % were achieved for covalently bound aerogels. Hybrid aerogel composites were successfully utilized as catalytic filters for the decomposition of nerve agent simulant and pesticide dimethyl-4-nitrophenylphosphate (DMNP), avoiding secondary pollution associated with MOF powder catalysis.

## Introduction

The increasing prevalence of agricultural and industrial pollutants in water bodies presents a major challenge to global water security,^1,2^ as water sources are frequently contaminated. Recently, more challenging pollutants like pharmaceuticals, pesticides, and metals (often found in concentrations from micro- to nanograms per liter) have been detected worldwide.^3,4^ The removal or breakdown of harmful chemicals is therefore a priority to safeguard clean water supplies. Since conventional wastewater treatment facilities are insufficient for removing these contaminants from water sources, it is necessary to develop new methods. However, many current solutions, such as biological treatment, ozonation, or reverse osmosis, often suffer from high costs and energy demands.^5,6^

Reticular chemistry offers a promising avenue to develop advanced materials, offering the principles of modular design for targeted applications such as water treatment.^7−9^ Metal–organic frameworks (MOFs) and covalent organic frameworks (COFs) are two classes of reticular materials that are especially attractive due to their crystalline, microporous structures and versatility,^10^ arising from the rational design of their building blocks^8^ or through postsynthetic modification (PSM).^11,12^ However, despite the vast library of over 100.000 different MOF structures currently cataloged at the Cambridge Crystallographic Data Centre (CCDC),^13^ their industrial and commercial applications remain constrained by the critical limitation of processability.

MOFs are usually obtained as fine powders, difficult to shape into useful forms without sacrificing their porosity and surface accessibility.^14−16^ However, direct use of MOF powders in water treatment introduces the additional requirement of MOF removal post-treatment and could lead to secondary water contamination.^17^ Attempts to utilize polymer binders to aid in MOF shaping or processing, such as for mixed matrix membranes, cannot avoid pore blockage, reducing mass transfer efficiency and hindering their practical usage. Novel methods to shape and process MOFs into macroscopic objects without compromising their intrinsic properties remain an unmet need.^18−20^

Recently, it has been shown by the Zamora group that it is possible to shape COFs into aerogels using simple sol–gel technology^21^ but their inert nature, while providing desired chemical stability, limits postsynthetic modifications to incorporate additional functionality. However, hybrid aerogels, which combine the structural integrity of one material with the functionality of another, are being developed for applications in gas separation,^22,23^ pollutant removal,^24,25^ water treatment,^14^ catalysis,^26^ or energy applications.^27,28^ In most cases, the two components in these hybrid materials interact with each other via van der Waals,^26,29^ ionic,^14^ or other noncovalent interactions.

The hybridization of MOFs and COFs into composite materials has sparked growing interest, particularly in core–shell structures that leverage the unique properties of both materials. Yet, the potential of COFs as microporous, processable binders for MOFs has remained largely unexplored. In this study, we introduce the novel use of COF matrices as porous MOF binders, overcoming the common challenge of pore blockage seen with traditional polymer binders, allowing for the creation of shapeable, catalytically active MOF/COF monoliths.^30−34^ The covalent integration of MOF particles into the COF aerogel matrix preserves both microporosity and structural stability, opening avenues for their utilization in real-world applications such as water treatment. As a proof of concept, these aerogels were used for simulated nerve agent methyl paraoxon degradation, demonstrating their potential for use in advanced water treatment applications.

## Results and Discussion

NH_2_–UiO-66 nanoparticles were synthesized according to a previously reported protocol due to its simplicity and predicted catalytic performance arising from induced defects through missing linkers.^35^ Successful synthesis was confirmed via powder X-ray diffraction (PXRD) (Figure S1). The morphology of the particles was examined by using field emission scanning electron microscopy (FE-SEM) (Figure S2), and the particle size was determined to be 103 ± 20 nm. MOF-808 nanoparticles were prepared via a previously reported room-temperature synthesis^36^ and were used due to their high catalytic activity in degrading nerve agent simulants.^37^ Successful synthesis was confirmed via PXRD and FE-SEM analyses (Figures S3 and S4). Particle size was determined to be 69 ± 7 nm via FE-SEM. Imine-based COF aerogels were synthesized by slightly modifying a procedure published previously.^21^ BTCA-TAPB COF was chosen as the model COF based on its superior pore stability, as reported by Zhu et al.^38^ and its ease of synthesis through Schiff-base formation. Successful COF formation was confirmed via PXRD, and the morphology of the microstructure was studied using FE-SEM (Figure S5), which showed good comparability to previously reported results.

### PSM of NH_2_–UiO-66 and MOF-808

**Figure 1:**
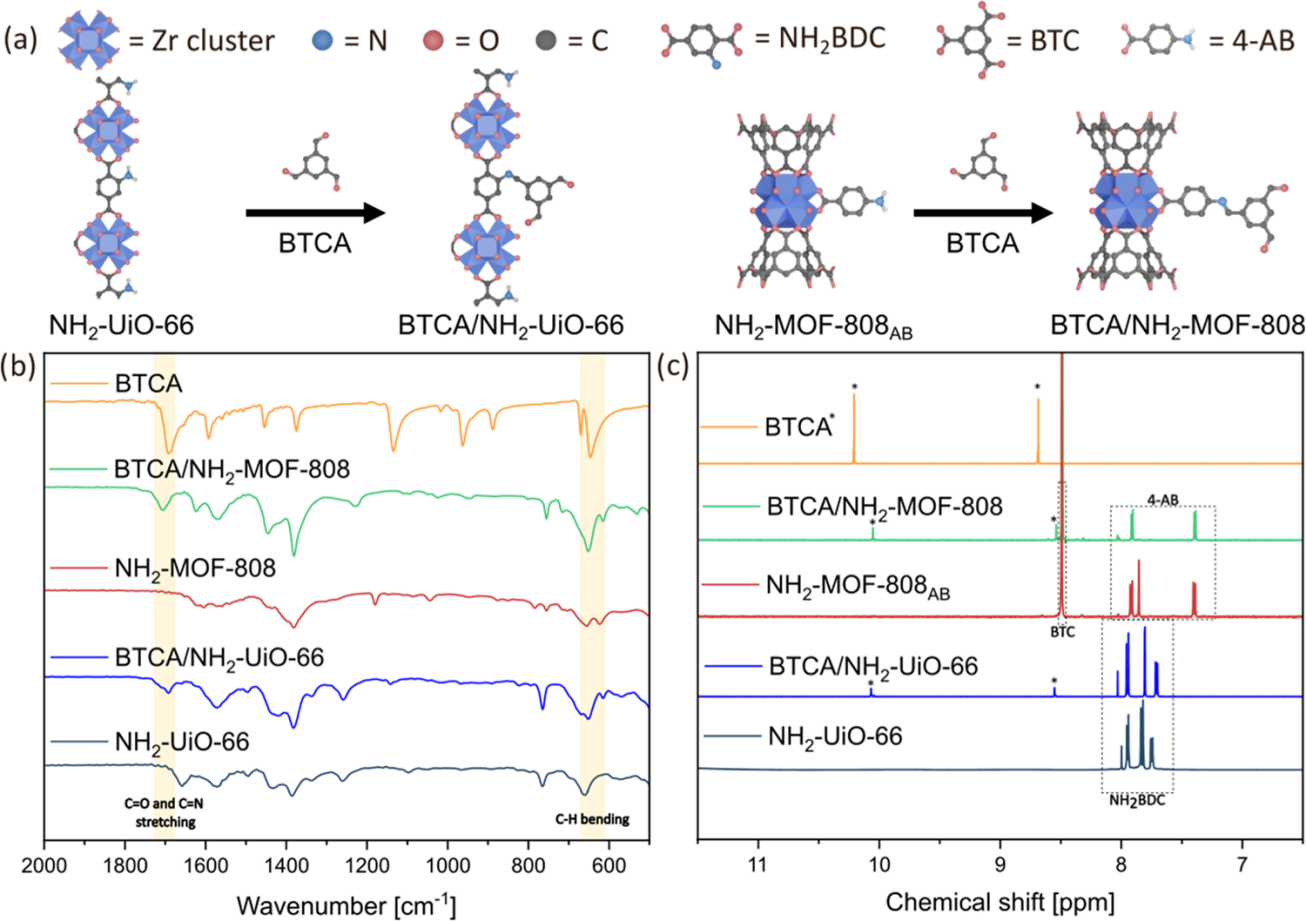
(a) Scheme of the PSM of NH_2_–UiO-66 and NH_2_-MOF-808 with BTCA, forming imine-functionalized MOFs; (b) FTIR spectra of NH_2_-MOFs, BTCA, and BTCA/MOF modifications; (c) ^1^H NMR spectra of digested MOFs before and after postsynthetic modification used to calculate the degree of functionalization.

Initial attempts to generate MOF/COF aerogels by dispersing the nanoparticles in benzene-1,3,5-tricarbaldehyde (BTCA) and 1,3,5-tris(4-aminophenyl)benzene (TAPB) solutions produced unsatisfactory results. The aging and washing steps of the gel synthesis resulted in the removal of MOF particles from the matrix, with a considerable decrease of MOF loading after supercritical CO_2_ (scCO_2_) drying (see Table 1 for details). This was attributed to the lack of sufficient binding between the MOF and the COF matrix, since the MOF particles could only be bound in the COF gel with weak physical forces, and the low density of the aerogel (0.02 g/cm^3^)^27^ was insufficient to physically retain nanoparticles of MOF during washing.^21,26,28,39^ To address these challenges, we explored the PSM of NH_2_–UiO-66 and NH_2_-MOF-808 with BTCA to evaluate the feasibility of integrating the MOF particles with the BTCA-TAPB COF via Schiff-base linkages. Previous studies have shown that modifying amino-MOFs via Schiff-base reactions typically requires long reaction times and high temperatures.^40,41^ However, we achieved the imine reaction by sonicating NH_2_–UiO-66 with BTCA in a 9:1 (v/v) acetic acid/water mixture at room temperature for just 1 h. After repeated washing of the functionalized NH_2_–UiO-66 with water, ethanol, and acetone (3× each) to remove excess unreacted BTCA, Fourier transform infrared spectroscopy (FTIR) and nuclear magnetic resonance spectroscopy (NMR) were used to observe the reaction. FTIR spectra of imine-functionalized NH_2_–UiO-66 (BTCA/NH_2_–UiO-66) showed the appearance of a band at 1690 cm^–1^ which can be attributed to both C=O and C=N stretching, with peaks at 1140 and 650 cm^–1^ indicating C–H in-plane and out-of-plane bending of BTCA, respectively (see Figure 1a). ^1^H NMR spectra of digested BTCA/NH_2_–UiO-66 confirmed the presence of BTCA, as the characteristic peaks of BTCA (8.59 and 10.08 ppm) were observed (see Figure 1b). Furthermore, the degree of functionalization of the postsynthetically modified MOFs was estimated to be 3–4%, consistent with previous MOF reports showing PSM of only the external surfaces of MOF crystals.^42^

**Table 1:** Loading Analysis of the MOF/COF Composites

**sample (wt % of MOF added)**	**amount of MOF added [%]**	**residual weight @800 °C [%]**	**MOF loading according to TGA [%]**
NH_2_–UiO-66	100	32.7	
NH_2_-MOF-808	100	41.8	
NH_2_–UiO-66/COF aerogel (75 wt %)	75	25.1	73.8
NH_2_–UiO-66/COF aerogel (75 wt %, no PSM)	75	15.3	40.3
NH_2_–UiO-66/COF aerogel (50 wt %)	50	18.8	52.3
NH_2_–UiO-66/COF aerogel (25 wt %)	25	11.0	25.6
NH_2_-MOF-808/COF aerogel (75 wt %)	75	32.3	74.8
NH_2_-MOF-808/COF aerogel (75 wt %, no PSM)	75	19.3	51.7
NH_2_-MOF-808/COF aerogel (50 wt %)	50	19.4	52.0
NH_2_-MOF-808/COF aerogel (25 wt %)	25	9.7	26.2
TAPB-BTCA COF	0	3.5	

Incorporation of MOF-808 into TAPB-BTCA aerogels was done in two steps due to the unsuccessful direct synthesis of MOF-808 nanoparticles bearing NH_2_ groups. First, postsynthetic ligand exchange (PSLE) of MOF-808, where benzene-1,3,5-tricarboxylic acid (BTC) is replaced with 5-aminoisophthalic acid (5-AI), was explored. It was found that by using *N*,*N*-dimethylformamide (DMF) as a solvent and varying the molar ratio of MOF-808:5-AI, the degree of linker exchange could be adjusted between 30 and 40% (Figure S6). For further experiments, NH_2_-MOF-808_AI_ (33% linker exchange) was used (see the Experimental Section for details).

As a comparison, MOF-808 nanoparticles were also modified postsynthetically by a nonstructural ligand exchange (NSLE) method, which is based on the exchange of nonstructural formate groups on MOF-808 secondary binding units for other monodentate carboxylic acids.^43,44^ 4-Aminobenzoic acid (4-AB) was employed for NSLE, with the degree of exchange monitored after a 12 h room-temperature PSM reaction via ^1^H NMR spectroscopy. 35% linker exchange was observed using a 10:1 molar ratio of 4-AB (Figure S7), and the resulting product, labeled as NH_2_-MOF-808_AB_, was utilized for further experiments.

Next, the reaction with BTCA was carried out under the same conditions as those for NH_2_–UiO-66. Interestingly, using NH_2_-MOF-808_AI_ resulted in no covalent binding with BTCA but instead led to degradation of the exchanged ligand, as shown by a steep reduction of 5-AI in ^1^H NMR spectra after the reaction (Figure S8). This was presumably due to the removal of the 5-AI from the MOF-808 structure during the reaction with BTCA, and this approach was therefore abandoned. We also carried out the reaction between NH_2_-MOF-808_AB_ and BTCA under the same conditions. FTIR (see Figure 1b) confirmed a successful addition of BTCA, with the appearance of an absorption band ranging from 1685 to 1735 cm^–1^ for BTCA/NH_2_-MOF-808 and from 1675 to 1730 cm^–1^ for BTCA/NH_2_–UiO-66, which corresponds to both C=O and C=N stretching. ^1^H NMR spectra (see Figure 1c) also confirmed the functionalization with the appearance of 8.54 and 10.05 ppm peaks attributed to the benzene ring and aldehyde hydrogens of BTCA, respectively. The degree of functionalization for BTCA/NH_2_-MOF-808, calculated as the ratio of BTCA to structural linkers via ^1^H NMR, was determined to be roughly 2.5%.

**Figure 2:**
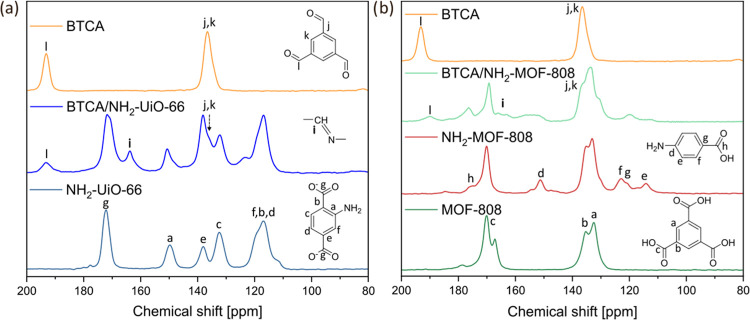
^13^C solid-state NMR of (a) NH_2_–UiO-66 and (b) NH_2_-MOF-808 before and after modification with BTCA (MOF-808 shown as a comparison).

Since digestion for ^1^H NMR of NH_2_–UiO-66 and NH_2_-MOF-808 with D_2_SO_4_ disrupted the imine bond between BTCA and NH_2_–UiO-66, solid-state ^13^C NMR was used to monitor the imine formation instead. After the PSM reaction, BTCA/NH_2_–UiO-66 showed a new peak at 193.2 ppm and a shoulder at 136.5 ppm corresponding to BTCA. Furthermore, a peak at 163 ppm was assigned to the carbon of the newly formed imine bond, thereby confirming successful reaction (see Figure 2a).^45^ A similar trend was observed for BTCA/NH_2_-MOF-808, where peaks originating from both 4-AB (151.3, 123.1, and 114.1 ppm), BTCA (190 ppm), and the imine (163 ppm) could be seen (see Figure 2b).

### Hybrid Aerogel Fabrication

We hypothesized that this simple modification could provide covalent binding of the NH_2_–UiO-66 and NH_2_-MOF-808 in the resulting TAPB-BTCA (COF) aerogel, resulting in improved particle retention during synthesis, aging, and subsequent applications. It was found that after combining the sonicated mixture of MOF and BTCA with TAPB solution (see Experimental Section for details), homogeneous gels formed after a few seconds. Importantly, no visible leaching of MOF or COF particles was observed, suggesting that the modification facilitated improved retention of the MOF nanoparticles in the COF gel. After scCO_2_ drying, hybrid MOF/COF aerogels were successfully produced. Solid-state ^13^C NMR showed that peaks corresponding to both the MOF and the COF components can be seen (Figure S9). However, due to the complexity of the sample and the resulting peak broadening, the imine peak formed between MOF and COF cannot be clearly observed in the solid-state spectra of hybrid aerogels.

The composition of the aerogels was analyzed by thermogravimetric analysis (TGA), revealing that stable MOF/COF composites with up to 75 wt % MOF loading could be achieved (see Table 1). Such a high level of MOF loading is beneficial for many practical applications, where performance-to-volume ratio is important. For samples where PSM was applied, MOF loading determined by TGA corresponded closely to the original amount of MOF added during synthesis, confirming that MOF particles were effectively retained in the matrix during the process. In contrast, attempts to prepare hybrid aerogels without prior PSM resulted in maximum MOF loading of 40.3 wt % for NH_2_–UiO-66/COF aerogels and 51.7 wt % for NH_2_-MOF-808/COF aerogels even with an initial MOF addition of 75 wt %, demonstrating the importance of covalent binding for creating stable hybrid MOF/COF composites with high MOF loadings. Details regarding the MOF loading calculation can be found in the SI (Figures S10–S12).

**Figure 3:**
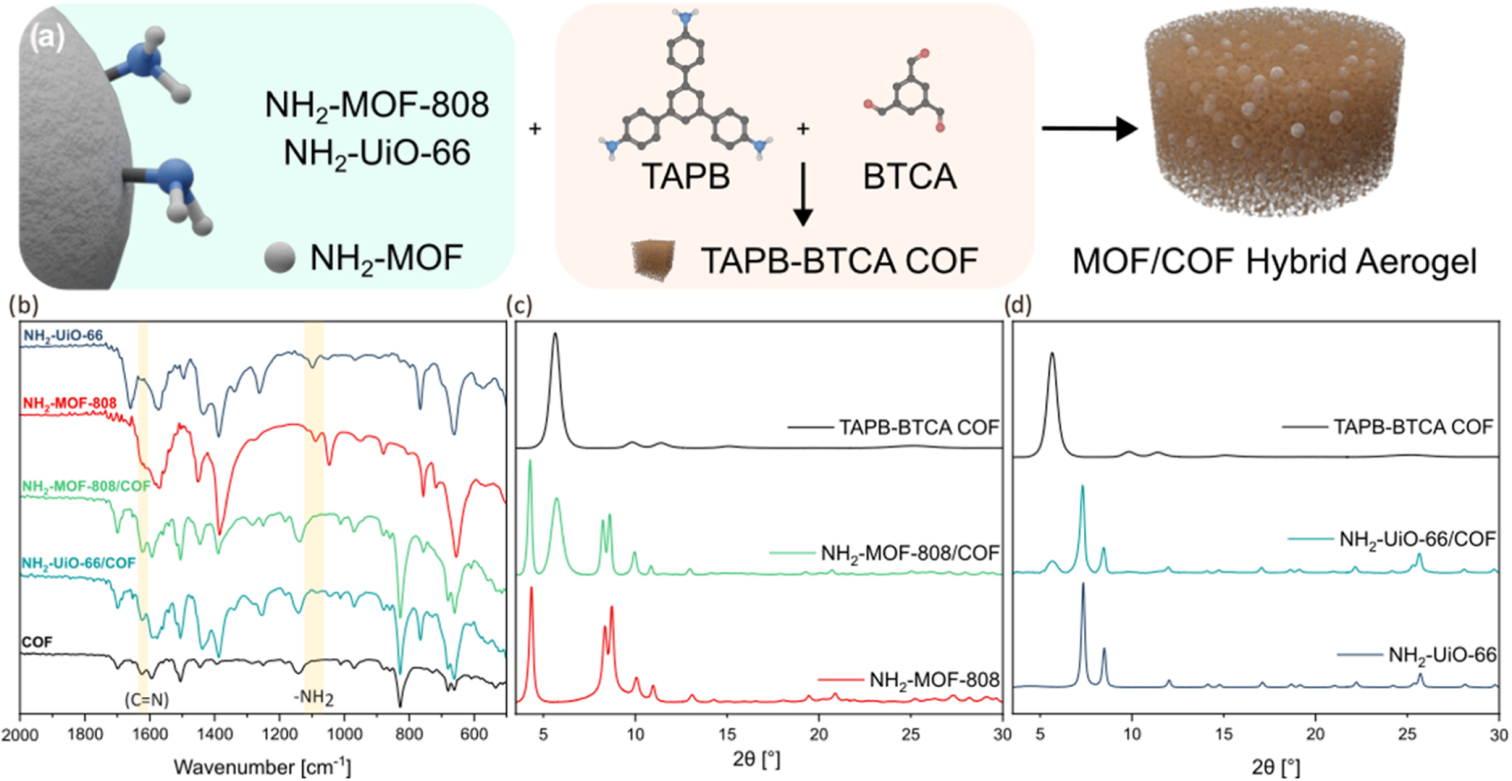
(a) Scheme of the MOF/COF hybrid aerogel preparation; (b) FTIR spectra of MOF powders and MOF/COF aerogels; (c, d) PXRD patterns of hybrid aerogels and their comparison with TAPB-BTCA COF and their microcrystalline forms for NH_2_-MOF-808/COF and NH_2_–UiO-66/COF.

FTIR spectroscopy of the hybrid aerogels (see Figure 3b) showed that the peaks at 1090 and 1087 cm^–1^ for NH_2_–UiO-66 and NH_2_-MOF-808, respectively, corresponding to the C–N stretch of the -NH_2_ group, decrease in the hybrid aerogel spectra, which we attribute to the conversion of these groups into imine bonds during the formation of the MOF/COF aerogel structure. The increase in the region from 1600 to 1650 cm^–1^, corresponding to the C=N stretches, in the MOF/COF samples supports the formation of the imine bonds at the MOF/COF interface and within the COF itself. The structures of the hybrid aerogels were confirmed by powder X-ray diffraction (PXRD) (Figure 3). The crystallinity of NH_2_–UiO-66 was retained during the preparation process, shown by characteristic peaks at 7.4 and 8.6° corresponding to the (111) and (200) reflections, respectively. Characteristic peaks of NH_2_-MOF-808 can be seen at 4.3, 8.2, and 8.6° corresponding to (111), (311) and (222) reflections. The crystallinity of the TAPB-BTCA COF was also confirmed with the existence of a 5.7° peak corresponding to the (100) plane, along with the peaks at 9.9 and 11.5° attributed to the (110) and (200) reflections.^21^ Compression tests (Figure S13) showed that the mechanical behavior of the NH_2_–UiO-66/COF and NH_2_-MOF-808/COF aerogels was enhanced in comparison with that of the TAPB-BTCA aerogels. The tested aerogels were compressed to 90% strain at a rate of 1 mm/min. Following compression, all samples partially recovered to approximately 15% of their original height without showing any signs of failure. This behavior is typical of elastomeric foams, which usually exhibit an initial Hookean region that allows for the calculation of the material’s Young’s modulus.^38^ The elastic modulus, calculated from the elastic (0–30% strain) region, was 2.9 kPa for the TAPB-BTCA aerogel, 13.7 kPa for NH_2_–UiO-66/COF aerogels, and 16.6 kPa for NH_2_-MOF-808/COF aerogels, indicating a significant improvement (Figure S14). The TAPB-BTCA aerogels prepared as a control were in good agreement with previously reported data by Martín-Illán et al.^21^ Notably, hybrid aerogels containing 75% either NH_2_–UiO-66 or NH_2_-MOF-808 showed twice the strength of TAPB-BTCA aerogels. The density of prepared aerogels was between 0.02 and 0.04 g/cm^3^ depending on the MOF loading (Figure S14)

**Figure 4:**
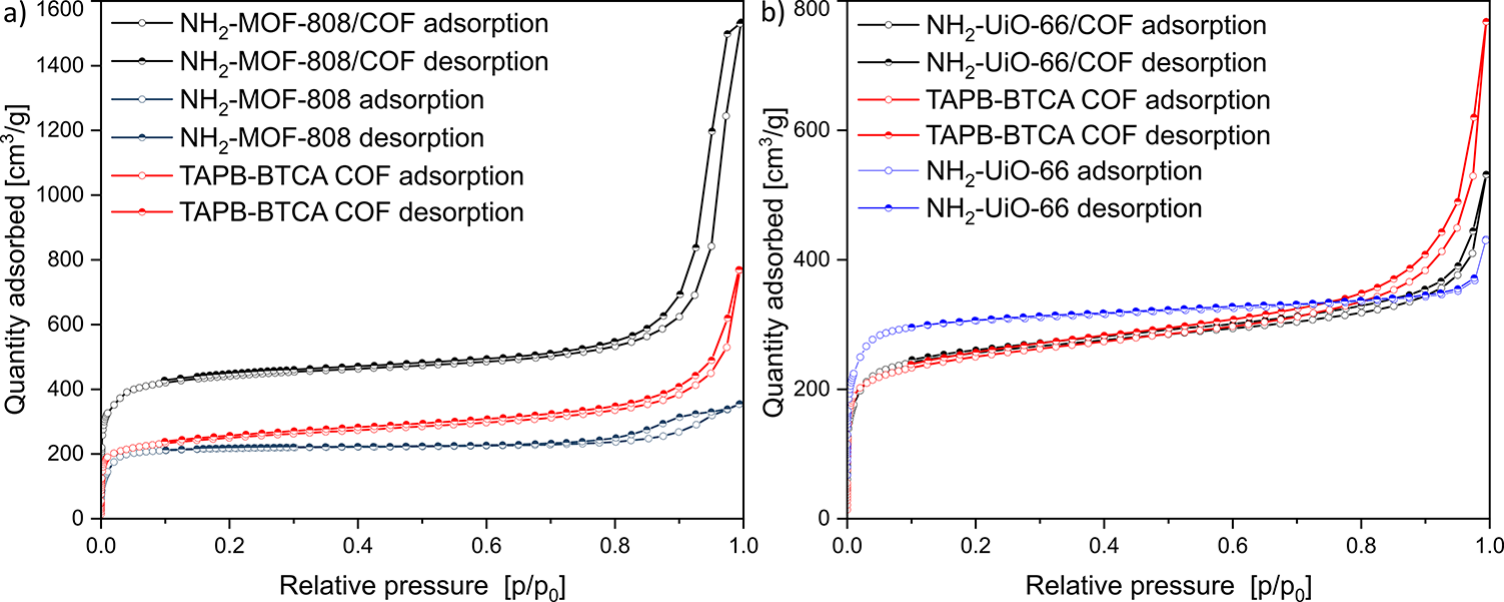
N_2_ adsorption–desorption isotherms of NH_2_-MOFs, TAPB-BTCA aerogel, and the hybrid MOF/COF aerogels showing increased porosity of (a) NH_2_-MOF-808/COF and retained porosity of (b) NH_2_–UiO-66/COF.

Importantly, the microporosity of both components is retained, as shown by the N_2_ physisorption at 77 K (see Figure 4). Isotherms of NH_2_–UiO-66, NH_2_-MOF-808, TAPB-BTCA COF, and the composite aerogel all show an initial steep increase, which corresponds to the adsorption of nitrogen into microcrystalline pores in the range of 1 to 2 nm,^19,33^ followed by a linear increase in adsorbed quantity over higher partial pressures for COF and MOF/COF samples, which suggest existence of larger pores. Interestingly, the BET surface area of NH_2_-MOF-808/COF aerogel (1790 m^2^ g^–1^) showed a 2-fold increase compared to the two constituent components (932 m^2^ g^–1^ for TAPB-BTCA COF, 941 m^2^ g^–1^ for NH_2_-MOF-808). The N_2_ sorption isotherm shows an increase in the micropore region, which follows the previously reported trend from Zamora and collaborators.^46^ The formation of new micropores could be due to the bridging of the MOF-808 Zr cluster to the COF through the 4-aminobenzoic acid, generating a MOF-COF interface whereby the geometry of the linkage allows sufficient room for N_2_ probe molecules during N_2_ sorption analysis. The increase in the mesoporous region suggests the formation of additional large pores around the MOF particles embedded in the COF matrix, and the presence of large pores is confirmed by the pore size distribution (Figure S15). When considering the NH_2_–UiO-66/COF aerogel, the obtained BET surface area of 987 m^2^ g^–1^ is on par with the BET values of NH_2_–UiO-66 (1229 m^2^ g^–1^) and the COF (932 m^2^ g^–1^), suggesting that the composite aerogel has retained available pores not hindered by monomers. The absence of a noticeable increase in the microporous region could be explained by the shorter and flexible bond between the NH_2_–UiO-66 moiety and the COF, separated only by a nitrogen atom, hindering the diffusion of N_2_.

As the TAPB-BTCA COF shows mesoporosity, but not NH_2_–UiO-66, the NH_2_–UiO-66/COF composite aerogel comprised approximately 75% of MOF and 25% of COF should show a significant decrease in mesoporosity to about 25% of the original COF. Instead, we observe an increase in adsorbed N_2_ between 0.1 and 0.8 P/P_0_, suggesting the formation of new mesopores at the interface of the MOF particles and the COF matrix.^47^ Moreover, the pore size distribution of the NH_2_–UiO-66/COF material shows the presence of large pores ranging from 4 to 40 nm, confirming the formation of these new pores (Figure S15). The overall increased porosity, both in the microporous and mesoporous regions could translate into improved properties for applications such as catalysis, which benefit from higher porosities.

**Figure 5:**
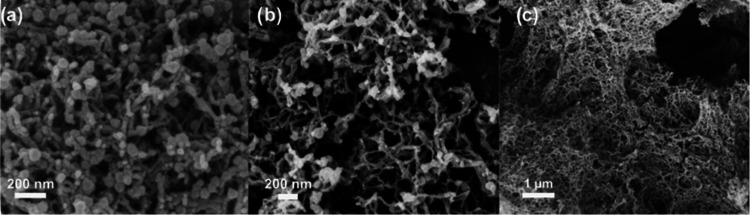
FE-SEM images of (a) NH_2_-MOF-808/COF aerogels; (b) NH_2_–UiO-66/COF aerogels, and (c) TAPB-BTCA aerogel showing uniform distribution of MOF nanoparticles in COF matrix.

FE-SEM images of aerogel samples showed a homogeneous dispersion of MOF particles in the TAPB-BTCA matrix (see Figure 5). Comparison of NH_2_-MOF-808/COF aerogels with different loadings (25, 50, 75%) confirmed even distribution of NH_2_-MOF-808 across all samples, showcasing the ability to easily control the loading of hybrid aerogels (Figure S16) while elemental mapping via energy-dispersive X-ray spectroscopy (EDS) confirmed uniform distribution of Zr, O, and C across the samples (Figure S17). Furthermore, STEM-EDS mapping was used to observe the hybrid structure of aerogels, confirming NH_2_-MOF-808_AB_ nanoparticles surrounded by TAPB-BTCA COF layers (Figure S18).

### Catalytic Degradation of DMNP

**Figure 6:**
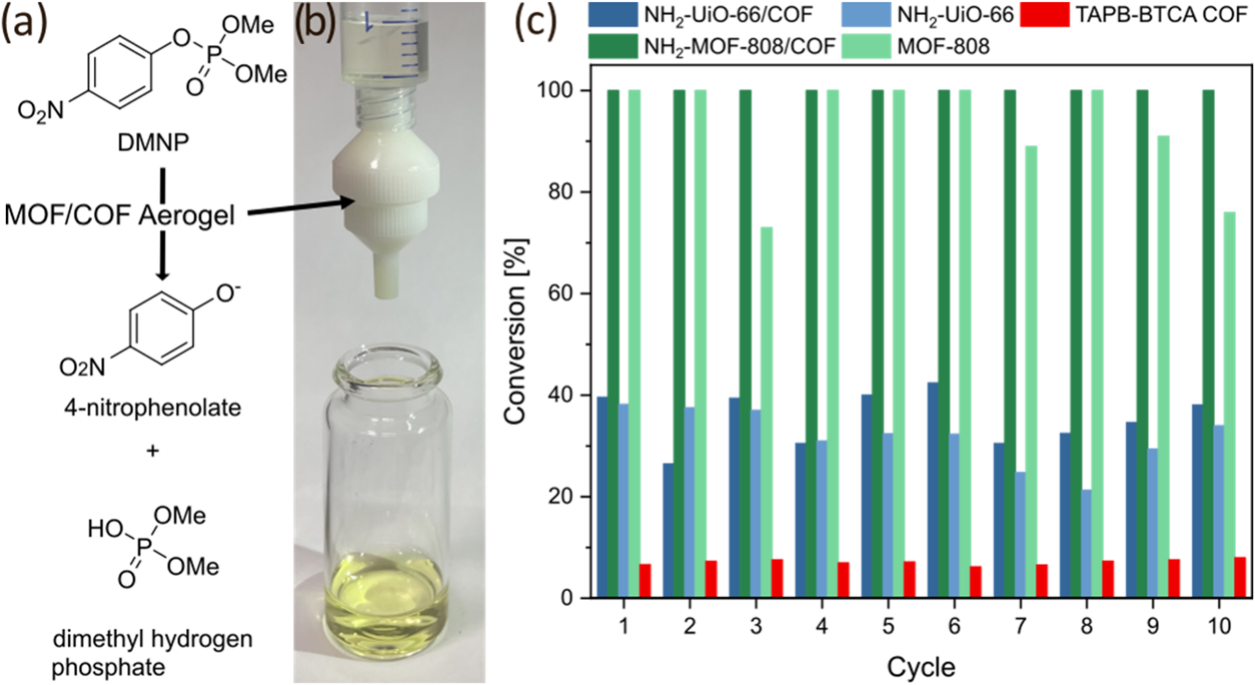
(a) Reaction scheme of DMNP degradation; (b) scheme of the filtration setup; and (c) conversion of DMNP over 10 cycles for hybrid aerogels, microcrystalline MOF-808, NH_2_–UiO-66, and TAPB-BTCA COF.

As a proof of concept for the application of the novel MOF/COF composite aerogels, they were employed as catalytically active filters for the removal of methyl paraoxon (dimethyl (4-nitrophenyl) phosphate, DMNP). Previous work has demonstrated that DMNP can be catalytically degraded via Zr-based MOFs.^45,46^ Several factors influence the degradation kinetics where the existence of structural defects, linkers with amine groups, and high porosity all improve degradation efficiency.^48^ It was proposed previously that the catalytically active sites are the aqua-ligating sites on the MOF node and that the mechanism of the reaction proceeds via zirconium oxy-hydroxide formed in situ.^49^ First, DMNP replaces one aqua ligand on the Zr_6_ node. Nucleophilic oxygen subsequently attacks the P=O bond, resulting in the release of 4-nitrophenolate and dimethyl hydrogen phosphate as products. Basic conditions are usually used to prevent poisoning of active sites.^50^ The product of DMNP degradation, dimethyl hydrogen phosphate, was measured via ^31^P NMR spectroscopy to monitor the reaction progress. Results showed that 25 mg of the NH_2_-MOF-808/COF aerogel can act as an active catalytic filter (Figure 6c), achieving full conversion of a 0.05 μL/mL DMNP (with 0.45 M *N*-ethylmorpholine) solution with a flow rate of 0.25 mL/min. The filtration was repeated 10 times using 1 mL of new solution (Figures S19 and S20). The filtration setup and comparison of the degradation using both hybrid aerogels, their microcrystalline powders, and TAPB-BTCA COF can be seen in Figure 6. In contrast, replicating the DMNP filtration with TAPB-BTCA aerogel using the same conditions results in very limited degradation, since the functional groups of the COF do not show catalytic activity toward this reaction. Also, catalysis of DMNP with both NH_2_–UiO-66 and NH_2_-MOF-808 powders lead to secondary pollution of the filtrate by MOF particles (Figure S21), despite the placement of a filter paper (Macherey-Nagel, quantitative) in the filter holder for MOF particle retention. This serves to highlight the importance of a supporting matrix that enables the retention and reuse of MOFs under applied conditions.

Due to the diverse reaction conditions and setups of MOF-based DMNP degradation catalysts in the literature, direct kinetic comparisons of previous catalysis results with our hybrid aerogels are not feasible. Nevertheless, Table 2 shows an overview of similar works on DMNP degradation. Previous works by Moon et al.^57^ and Ma et al.^58^ utilize MOF-808 powder suspended in a syringe filter and MOF-808@fibrous bacterial cellulose aerogel, respectively, to show that the high activity of MOF-808 can be utilized for DMNP degradation in a flow setup. It must be noted that when comparing flow setups, contact times of DMNP with the catalyst cannot be directly determined from the flow rates of previous works, since they are dependent on the reactor geometry. As can be seen from Table 2 and Figure 6, the high catalytic activity of our NH_2_-MOF-808/COF aerogel and the correspondingly lower activity of our NH_2_–UiO-66/COF aerogel are in accordance with previously reported observations.^48−50,52,59^

**Table 2:** Overview Table of Reported MOFs for DMNP Degradation and the Results from This Work

**material**	**reaction time or flow rate**	**state of the catalyst**	**type of system**	**refs**
UiO-66	170 min (84% conversion)	powder	in solution (batch)	(51)
NH_2_–UiO-66	35 min (75% conversion)	powder	in solution (batch)	(49)
MOF-808	26 min	powder	in solution (batch)	(49)
NU-1000	60 min	powder	in solution (batch)	(48)
Im@MOF-808	35 min	powder	in solution (batch)	(37)
PPFM-1 (NH_2_–UiO-66/PFF)	30 min	MOF@fiber	in solution (batch)	(52)
PA-6@NH_2_–UiO-66	60 min	MOF@fiber	in solution (batch)	(53)
PAN@Zr(OH)_4_@MOF-808	8 min	MOF@fiber	in solution (batch)	(54)
UiO-67/Ce(OH)_4_@PIM-1	15 min	MOF@membrane	in solution (batch)	(55)
MOF-808@CNF-PQ	240 min	MOF@membrane	in solution (batch)	(56)
MOF-808	0.1 mL/min	powder	flow setup	(57)
FMOF-808-NA1	0.1 mL/min	MOF@aerogel	flow setup	(58)
NH_2_-MOF-808/COF	0.25 mL/min	MOF@aerogel	flow setup	this work
NH_2_–UiO-66/COF	0.25 mL/min (35% conversion)	MOF@aerogel	flow setup	this work

## Experimental Section

### UiO-66 and NH_2_–UiO-66 Synthesis

UiO-66 and NH_2_–UiO-66 nanoparticles were synthesized according to previous research by Katz et al.^35^ Briefly, a 20 mL cylindrical vial was loaded with 125 mg of ZrCl_4_, 5 mL of DMF, and 1 mL of conc. HCl. Afterward, the vial was sonicated for 15 min or until the full dissolution of ZrCl_4_. To a second vial, 10 mL of DMF and the linker (123 mg of terephthalic acid for UiO-66; 134 mg of 2-aminoterephthalic acid for NH_2_–UiO-66) were added before being sonicated for 20 min. The contents of the two vials were mixed and held at 80 °C overnight. The resulting solid was collected using centrifugation (10 000 rpm, 10 min) and washed twice with DMF and twice with EtOH.

### MOF-808 Synthesis

MOF-808 nanoparticles were synthesized in accordance with the room-temperature approach reported by Dai et al.^36^ First, Zr_6_ oxo-clusters were prepared by heating a mixture of 5 g of ZrCl_4_, 12.5 mL of isopropanol, and 7.5 mL of acetic acid at 120 °C for 1 h. The obtained white powder was recovered by centrifugation, washed with acetone twice, and dried under vacuum at room temperature. Next, Zr_6_ oxo-clusters (0.6 g) were stirred in 0.75 mL of formic acid and 1.25 mL of H_2_O until the solution became colorless. Afterward, 1,3,5-benzenetricarboxylic acid (BTC) (150 mg) was added, and the resulting solution was stirred overnight. The resulting nanoparticles were collected by centrifugation (14 500 rpm, 5 min), washed with H_2_O and EtOH, and dried in a vacuum at room temperature, yielding 0.55 g of white powder.

### NH_2_-MOF-808 Synthesis

#### NH_2_-MOF-808 by Postsynthetic Ligand Exchange (PSLE)

NH_2_-functionalized MOF-808 was first prepared utilizing PSLE by adopting a previously reported procedure.^60^ 90–150 mg of 5-aminoisophthalic acid was dissolved in 10 mL of either MeOH or DMF. 100 mg of MOF-808 was added to the solution and subsequently dispersed via sonication for 5 min. Afterward, the mixture was stirred at 25 °C for 90 min to 24 h. Resulting NH_2_-MOF-808 was collected via centrifugation, washed repeatedly with H_2_O, EtOH, and acetone, and dried under vacuum to obtain 92 mg yellow powder.

#### NH_2_-MOF-808 by Formate Exchange

MOF-808 was modified postsynthetically via formate exchange^43^ using 4-aminobenzoic acid to obtain NH_2_-MOF-808. 200 mg of 4-aminobenzoic acid was dissolved in 10 mL of DMF. 200 mg of MOF-808 was added to the solution and subsequently dispersed via sonication for 5 min. Afterward, the mixture was stirred at 25 °C for 12 h. The resulting NH_2_-MOF-808 was collected via centrifugation (14 500 rpm, 5 min) and washed repeatedly with H_2_O, EtOH, and acetone to obtain a yellow powder.

### TAPB-BTCA COF Synthesis

The synthesis was adapted from a previous publication.^21^ Monomer solutions were prepared by dissolving 1,3,5-benzene tricarbaldehyde (BTCA) in a mixture of 90 vol % of acetic acid and 10 vol % of Milli-Q water (3.25 mg BTCA/mL) and separately dissolving 1,3,5-Tris(4-aminophenyl)benzene (TAPB) in the same solvent ratio (7 mg/mL; 9/1 v/v AcOH/H_2_O). Both solutions were sonicated until completely dissolved. After mixing equal amounts of the two solutions, a yellow gel was quickly formed. Prepared gels were aged in pure acetic acid for 5 days, solvent-exchanged with tetrahydrofuran and subsequently ethanol (3× each in 2-h intervals). Washed gels were scCO_2_ dried using supercritical CO_2_ (scCO_2_) drying from EtOH to obtain aerogels.

### MOF/COF Synthesis

Monomer solutions described above were used with increased concentrations of the BTCA (4 mg/mL) and TAPB (8.8 mg/mL). A calculated amount of MOF particles was weighed, added to the BTCA solution, and sonicated for 1 h. Afterward, a solution containing an equal molar amount of TAPB was quickly added and mixed to obtain an orange gel (gels in volume of 1–4 mL were typically prepared, resulting in 13–55 mg aerogels after drying). Aging, washing, and drying steps were identical to the procedure for a typical COF synthesis described above. For samples with no postsynthetic modification of MOF particles with BTCA, MOF particles were dispersed in the TAPB solution before COF formation instead, reducing the reaction between BTCA and MOF to a minimum.

Degradation studies were performed in a 5 mL Luer-lock syringe connected to a 13 mm Swinney filter holder (Cytiva). For aerogel samples, 25 mg of either NH_2_-MOF-808/COF or NH_2_–UiO-66/COF (75% MOF loading) was used; an equal amount of MOF powder (18.5 mg) was used as a comparison for filtration using microcrystalline MOFs. Due to the powder nature of microcrystalline MOFs, samples were loaded into the filter holder with a filter paper (Macherey-Nagel, quantitative). The initial concentration of DMNP was 0.05 μL/mL, buffered with 0.45 M *N*-ethylmorpholine with a solvent mixture of 9:1 (v/v) H_2_O:D_2_O in order to directly measure the samples via ^31^P NMR afterward. The reaction was stopped directly after the filtration by adding a few drops of HCOOH (reagent grade, Sigma). The flow rate was controlled with a syringe pump (Nordson EFD 8000 Ultra).

## Analytical Methods

### Powder X-ray Diffraction

Powder X-ray diffraction (PXRD) measurements were carried out on an Empyrean Panalytical. The Anode Material is Cu, Step Size [°2θ] is 0.0130, Generator Settings: 40 mA, 45 kV, measurement was performed at ambient temperature.

### N_2_ Sorption Measurements

N_2_ sorption measurements were carried out on a Quantachrome iQ2 instrument (Anton Parr). The samples were activated at 150 °C for 16 h, and analyses were performed at 77 K. The surface area was calculated using the Brunauer–Emmett–Teller (BET) method.

### Thermogravimetric Analysis

Thermogravimetric analysis (TGA) was done on a TGA/DSC 3+ STARe Thermal Analysis System Jupiter under air. Samples were heated from 50 to 800 °C at 10 °C/min.

### Supercritical CO_2_

Supercritical CO_2_ drying was performed on a Leica CPD300 instrument provided by the CIUS core facility, Faculty of Biology, University of Vienna.

### Infrared Spectroscopy

Fourier transform infrared (FTIR) spectroscopy was carried out in a Bruker TENSOR 37 in an attenuated total reflectance (ATR) mode with the following settings: 4 cm^–1^ resolution, scan rate of 16 scans, measurement range of 450–4000 cm^–1^.

### Nuclear Magnetic Resonance

Nuclear magnetic resonance (NMR) spectra were recorded on either a Bruker BioSpin AV III 600 (^1^H NMR: 600.25 Hz, ^13^C NMR: 150.93 Hz) or Bruker BioSpin AV neo 500 (^1^H NMR: 500.32 Hz, ^13^C NMR: 125.81 Hz, ^31^P NMR: 202.44 Hz) provided by the NMR Center, Faculty of Chemistry, University of Vienna. The residual solvent peaks were used as a reference. The resulting spectra were processed and analyzed by using TopSpin 4.3.0.

### UV–vis Spectroscopy

UV–vis spectroscopy was measured on an Agilent Cary 60 UV–vis spectrometer.

### Mechanical Properties

Compression behavior was monitored with an in-house built setup in a DEBEN microtester with a 2 kN load cell. A rate of 1 mm/min, sampling interval of 500 ms, and 50× gain were used to record data during the measurement.

### Electron Microscopy and Energy-Dispersive X-ray Spectroscopy

Electron microscopy (EM) measurements were performed at the electron microscopy center at the Electron Microscopy Facility of IST Austria. High-angle annular dark-field scanning transmission electron microscopy (HAADF-STEM) and energy-dispersive X-ray (EDX) spectroscopy measurements were carried out using a S/TEM Jeol JEM2800 with an accelerating voltage of 200 kV. The EM micrographs were collected with a CMOS TEM camera TemCam-XF416, and EDX result was gathered by an EDX detector Jeol Centurio, which is a large solid angle silicon drift detector with 100 mm^2^ active area for ultrafast elemental mapping of S/TEM samples. All samples were prepared by drop-casting onto 200-mesh copper grids coated with a carbon film (Shenzhen Rigorous Technology Co., Ltd.). Subsequently, the samples were dried in an oven at 70 °C before usage.

## Conclusions

In this work, we have developed an effective method to prepare novel, processable, and monolithic MOF/COF hybrid aerogel composites. By utilizing covalent binding between the two components, the retention of MOF particles in the COF matrix was greatly improved, which allowed for the preparation of MOF/COF hybrid aerogels with up to 75% MOF loading. Detailed analysis of hybrid MOF/COF aerogels confirmed retained crystallinity of both components and increased microporosity, which enables exceptional substrate accessibility to the MOF-based catalytic sites. As a proof-of-concept application, aerogels were used for the effective degradation of the nerve agent simulant and pesticide DMNP in a filtration setup, where MOF nanoparticle retention by the COF binder removed the need for additional filtration or centrifugation of the filtrate. The reaction was studied via ^31^P NMR spectroscopy, with aerogels showing exceptional stability over 10 cycles. Results show great potential toward the practical utilization of MOF/COF composites in advanced applications.

## Supporting Information Available

Additional detailed experimental results and characterizations (e.g., FE-SEM, PXRD, NMR, TGA, mechanical tests) supporting the manuscript findings (PDF)
