# Supplementary material for: Covalently Bound MOF/COF Aerogels as Robust Catalytic Filters for Rapid Nerve Agent Decomposition
Source: ACS Appl Mater Interfaces. 2025 Mar 3;17(10):15938–47. doi: 10.1021/acsami.4c19759 (PMC11912193; doi:10.1021/acsami.4c19759)
Supplement: Supplementary file 1 — am4c19759_si_001.pdf [file am4c19759_si_001.pdf]

Supporting information

Covalently bound MOF/COF aerogels as robust catalytic filters for rapid nerve agent decomposition

Martin Sahuľ(a,b), Youven Benseghir(a), Tanja Eder(a), Flora Schöfbeck(a,b), Lingcong Ge(b,c), Dániel Hetey(a), Michael R. Reithofer(c), Jia Min Chin*(a)*

(a) Institute of Functional Materials and Catalysis, Faculty of Chemistry, University of Vienna, Währinger Str. 42, 1090 Vienna, Austria;

(b) Vienna Doctoral School in Chemistry (DoSChem), University of Vienna, Währinger Str. 42, 1090 Vienna, Austria;

(c) Institute of Inorganic Chemistry, Faculty of Chemistry, University of Vienna, Währinger Str. 42, 1090 Vienna, Austria

*Corresponding Authors:

Jia Min Chin - Department of Functional Materials and Catalysis, University of Vienna, Währinger Straße 42, 1090 Vienna, Austria; E-mail: jiamin.chin@univie.ac.at

Michael Reithofer - Institute of Inorganic Chemistry, University of Vienna, Währinger Straße 42, 1090 Vienna, Austria; E-mail: michael.reithofer@univie.ac.at

Supporting information

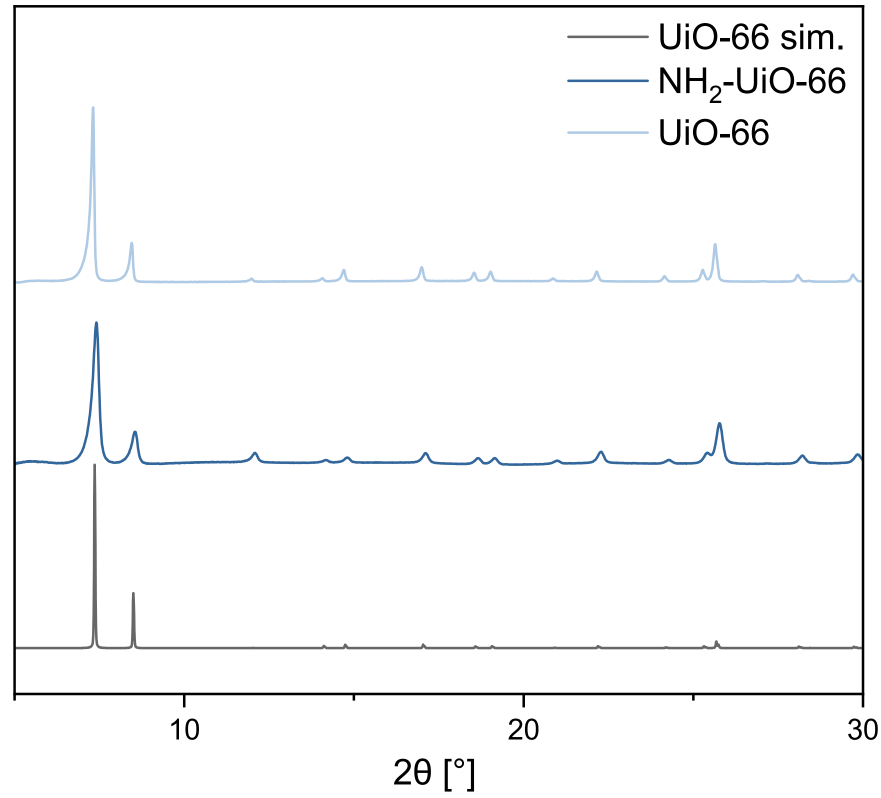

S1: PXRD of as-synthesized UiO-66 and NH_2-UiO-66.

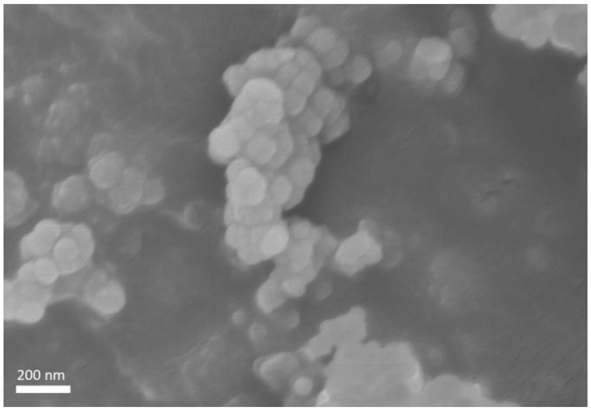

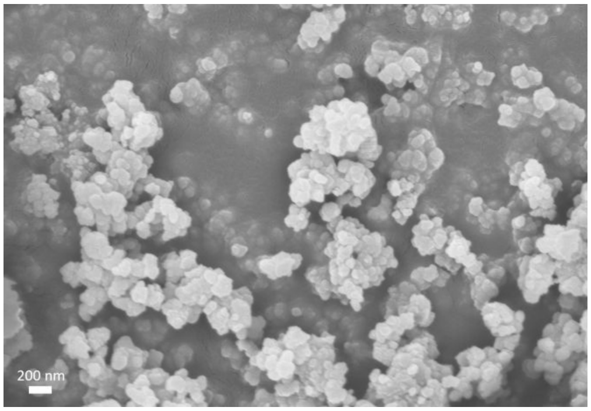

S2: FE-SEM Images of UiO-66 (left) and NH_2-UiO-66 (right).

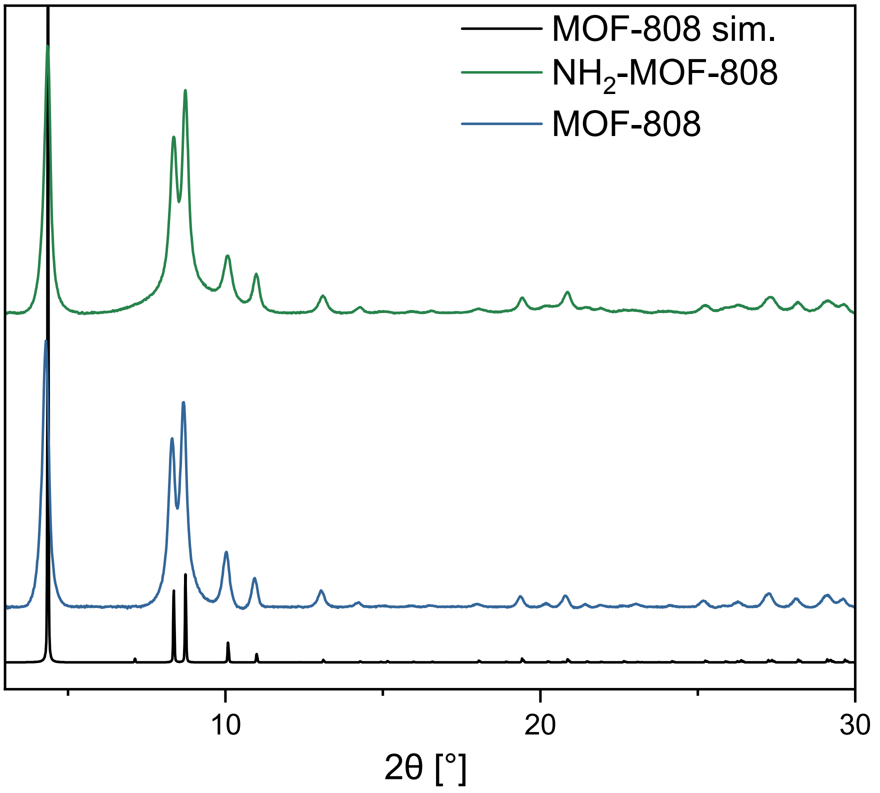

S3: PXRD of MOF-808 nanoparticles and NH_2-modified MOF-808 nanoparticles.

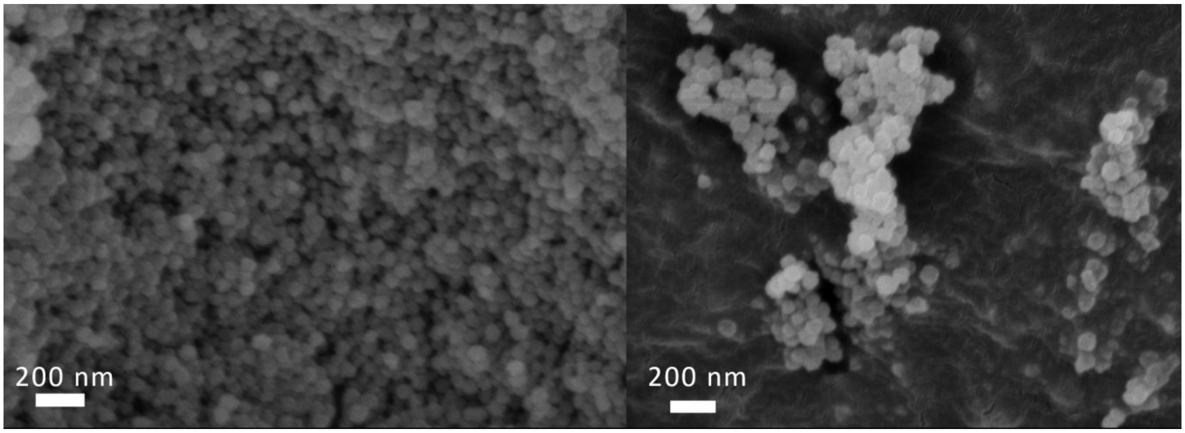

S4: FE-SEM Images of MOF-808 nanoparticles.

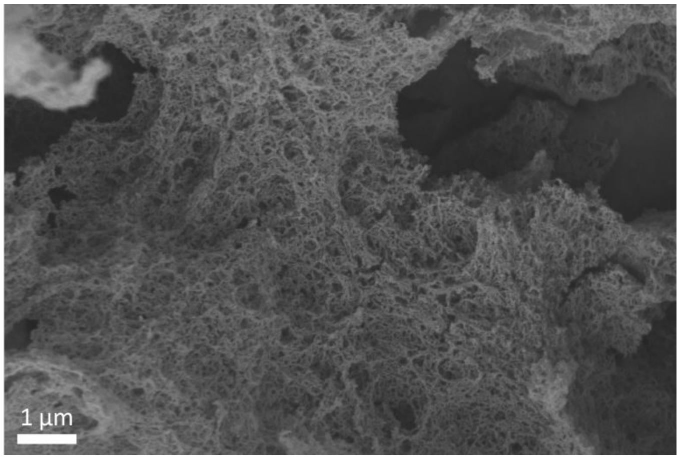

S5: FE-SEM image of TAPB-BTCA COF.

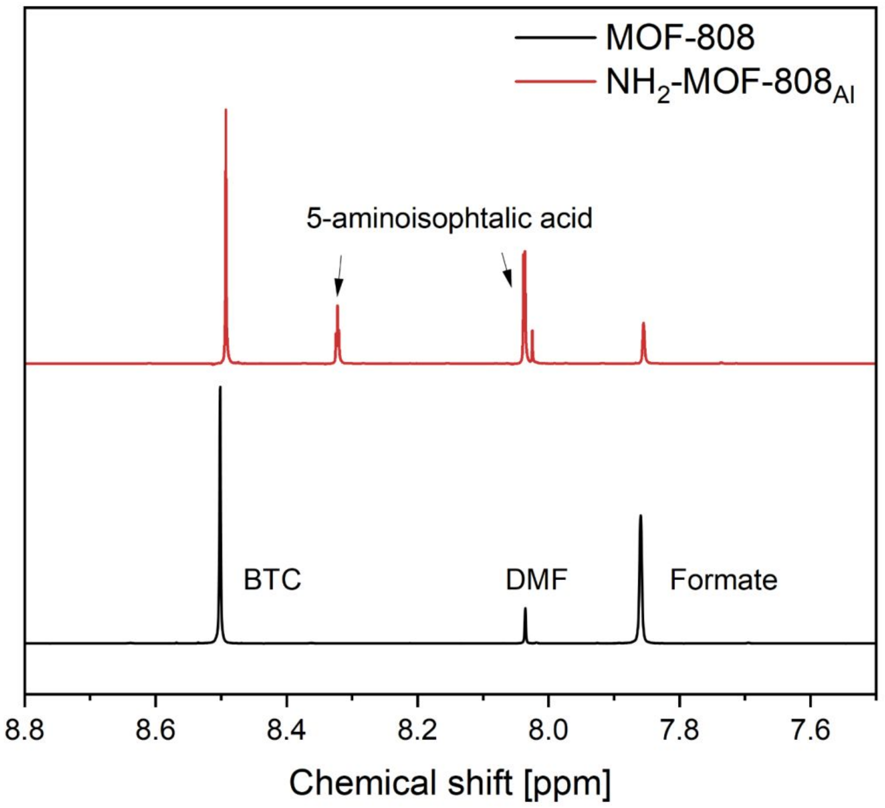

S6: NMR of 5-AI modified MOF-808.

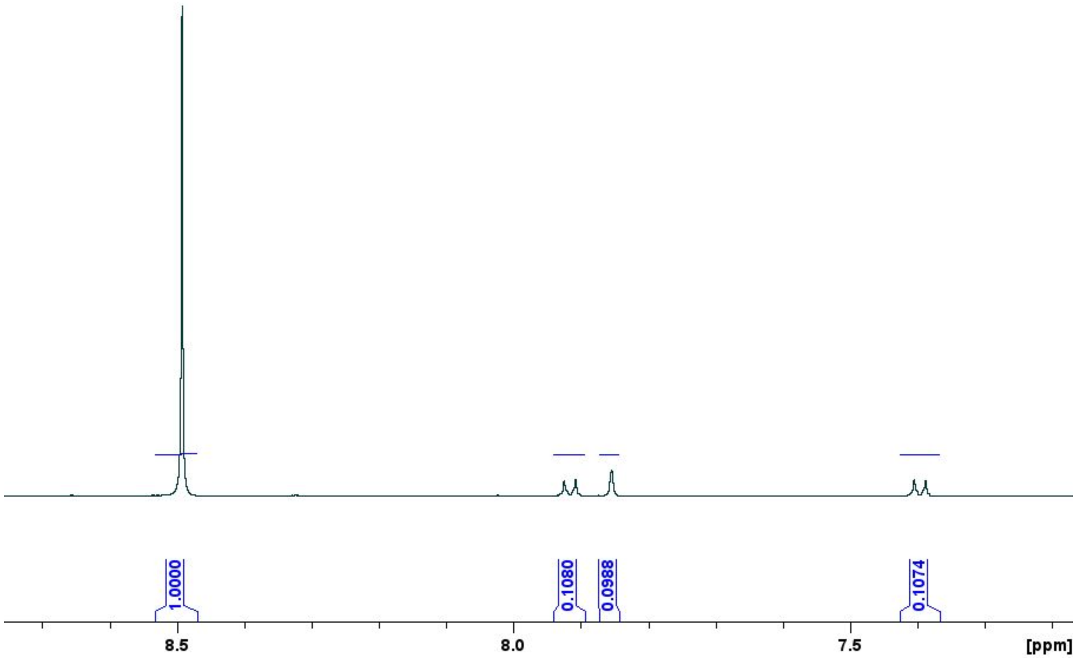

S7: Integration of ^{1}H NMR spectrum of 4AB-MOF-808 modification, showing a 65:35 ratio between formate and 4-AB. (This spectrum is also plotted in Figure 1c in red).

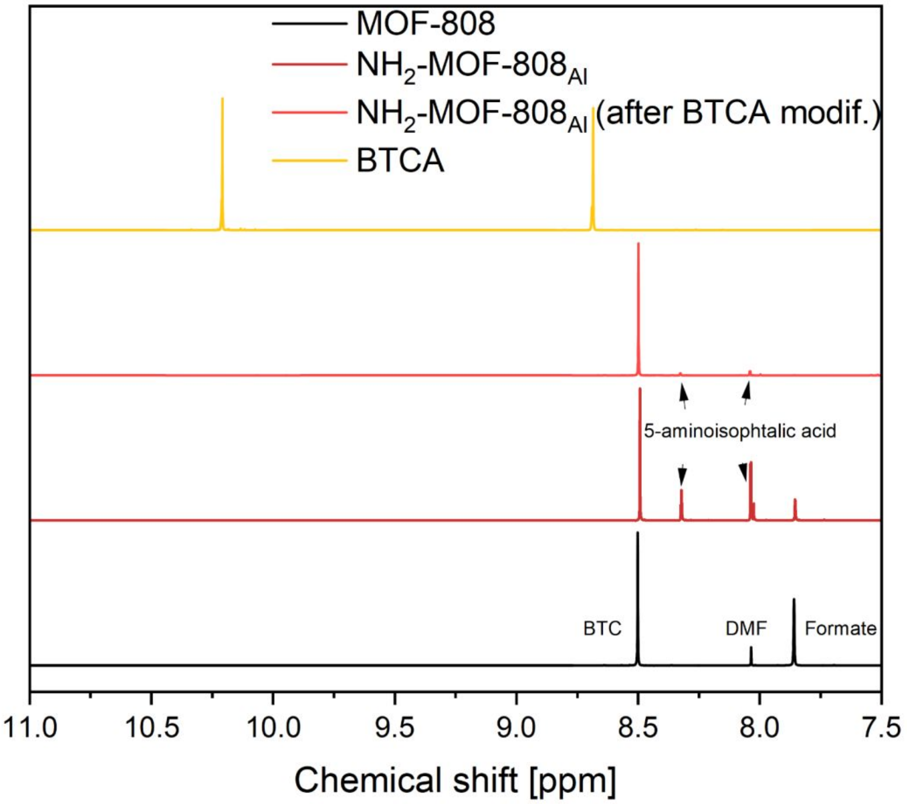

S8: MOF-808 (black), NH_2-MOF-808_{AI} (red), BTCA (yellow) and NH_2-MOF-808_{AI} after reaction with BTCA showing that only 9 % of 5-AI remains and that no bound BTCA was detected.

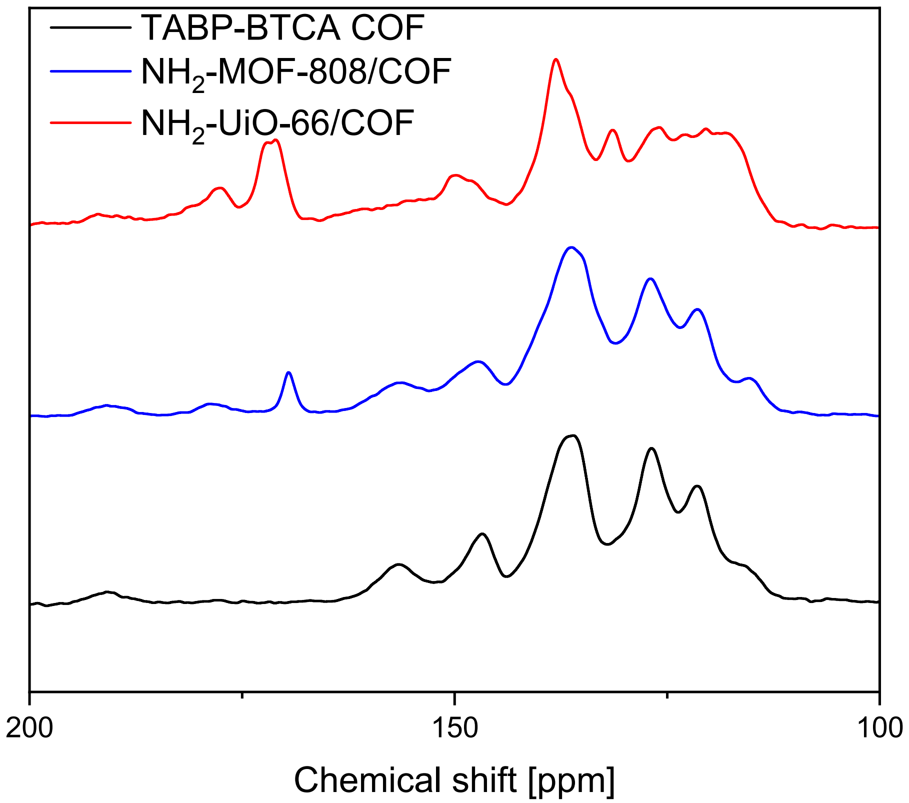

S9: solid-state ^{13}C NMR of hybrid MOF/COF aerogels in comparison to TAPB-BTCA aerogel.

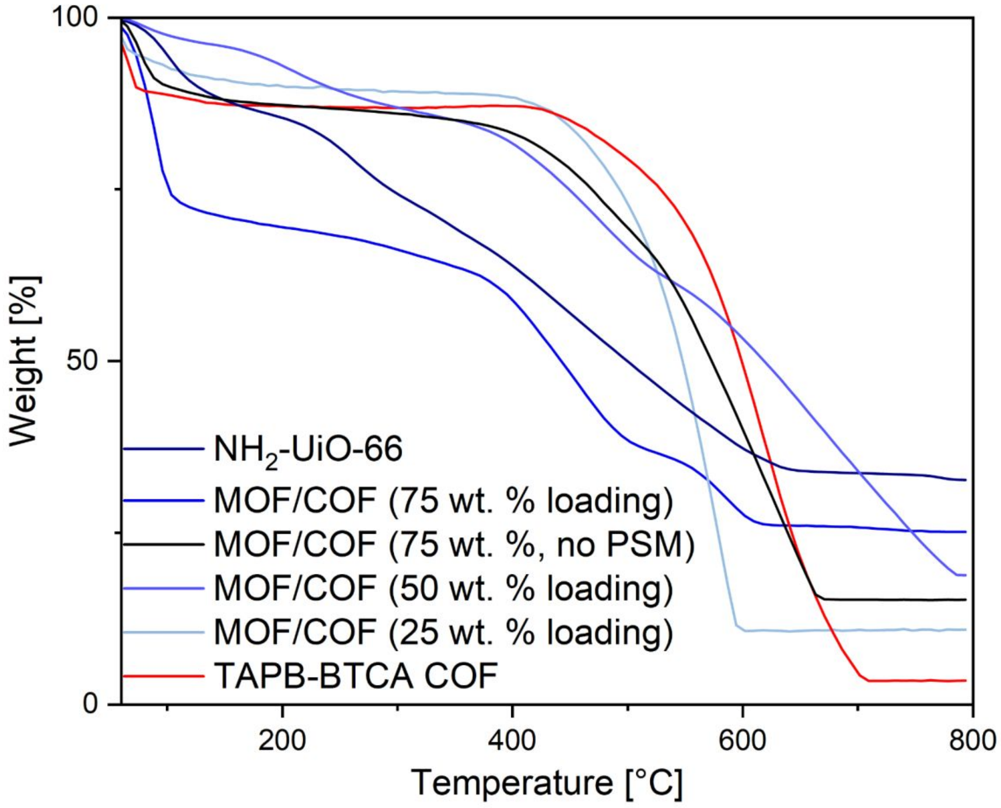

S10: TGA curves of NH_2-UiO-66/COF composites.

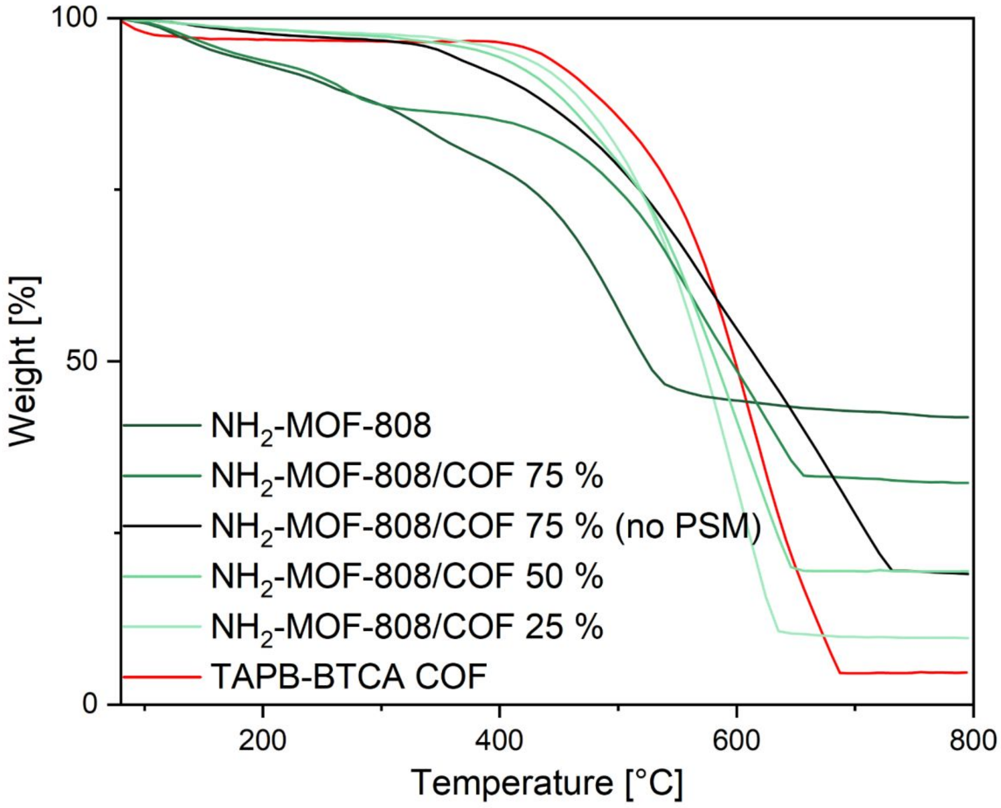

S11: TGA curves of NH_2-MOF-808/COF composites.

$$m\%_{MOF@800} \cdot \omega + m\%_{COF@800} \cdot (1 - \omega) = m\%_{Sample@800}$$

,where $m\%_{MOF@800}$ is equal to the residual weight of MOF powder at 800 ˚C, $m\%_{COF@800}$ is residual weight of TAPB-BTCA COF at 800 ˚C, $m\%_{Sample@800}$ is the residual weight of MOF/COF composite at 800 ˚C and ω is the resulting MOF loading of the MOF/COF composite

S12: Calculation of MOF loading.

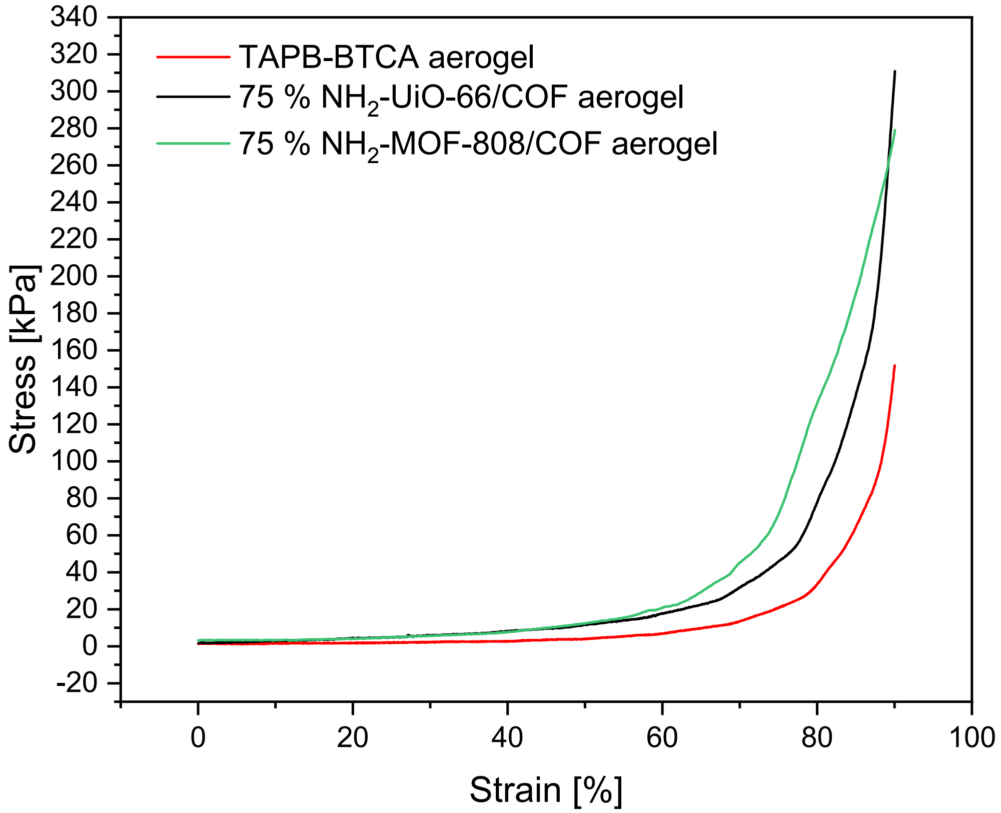

S13: Compression testing of MOF/COF composites and TAPB-BTCA aerogel.

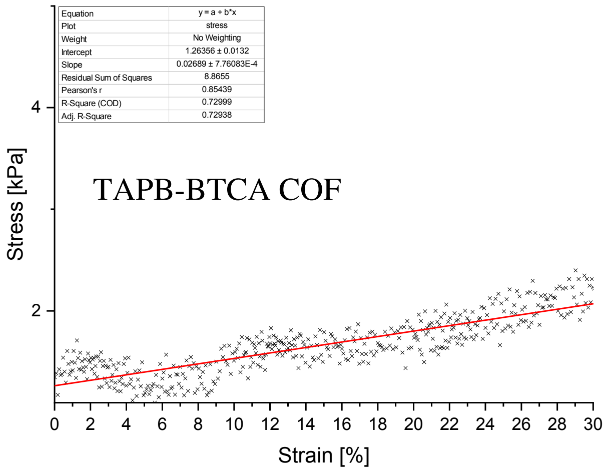

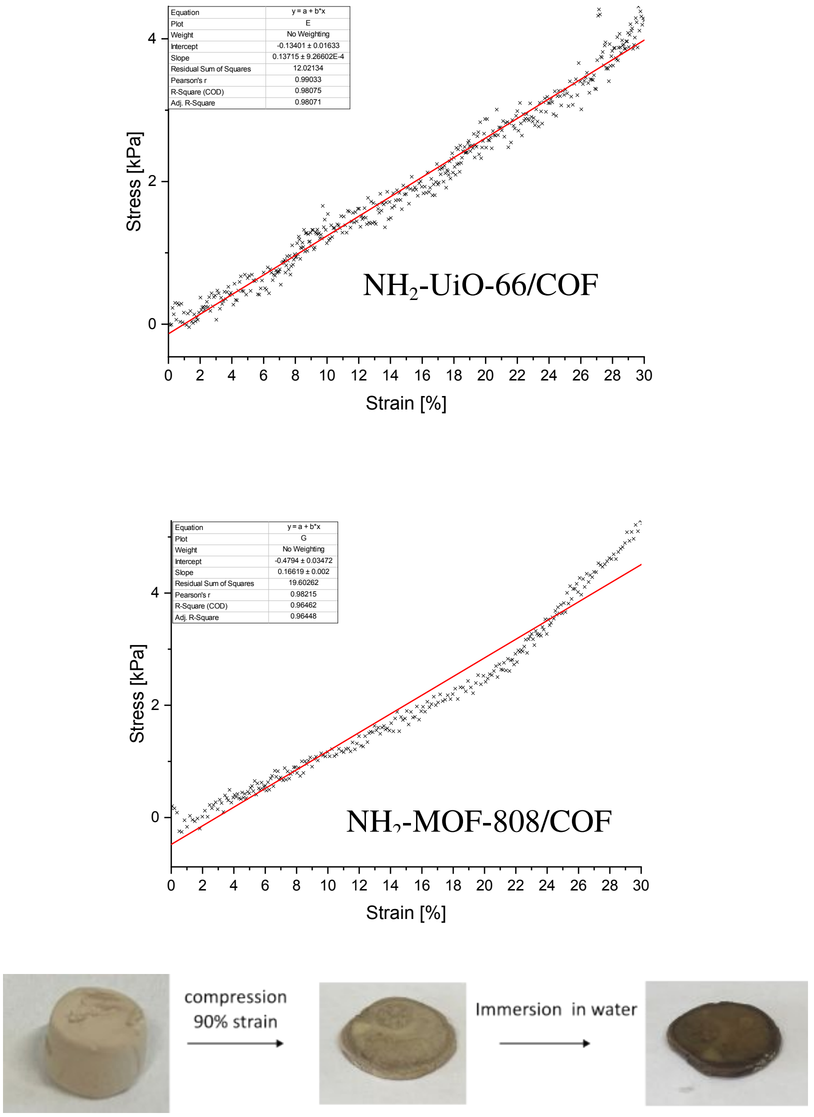

S14: Linear fitting of the elastic strain region (0 – 30 %) used to calculate apparent Young modulus (top); Images showing stability of the MOF/COF aerogels after compression and water immersion (bottom).

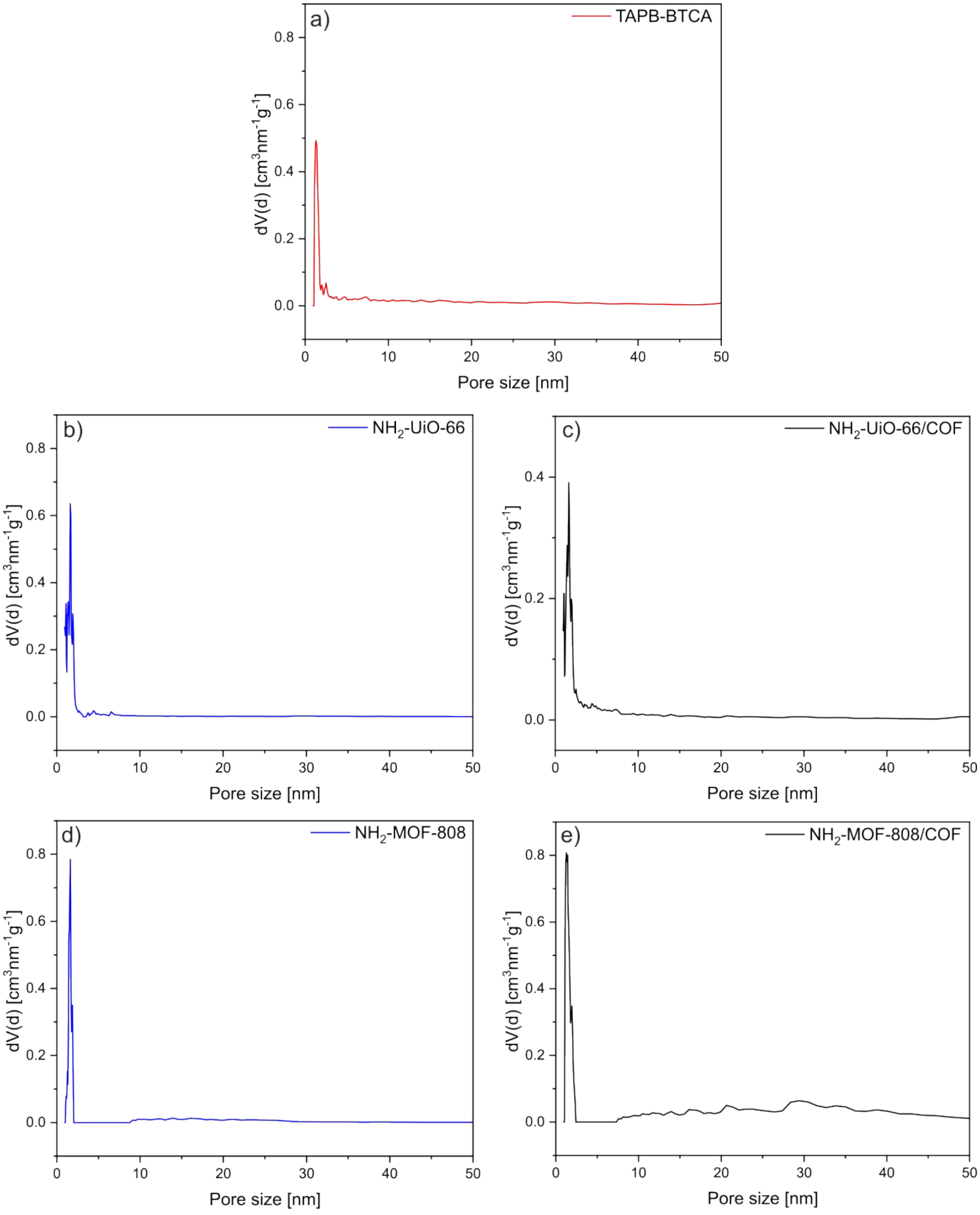

S15: Pore size distribution of (a) TAPB-BTCA COF; (b,d) MOF powders and (c,e) MOF/COF composites.

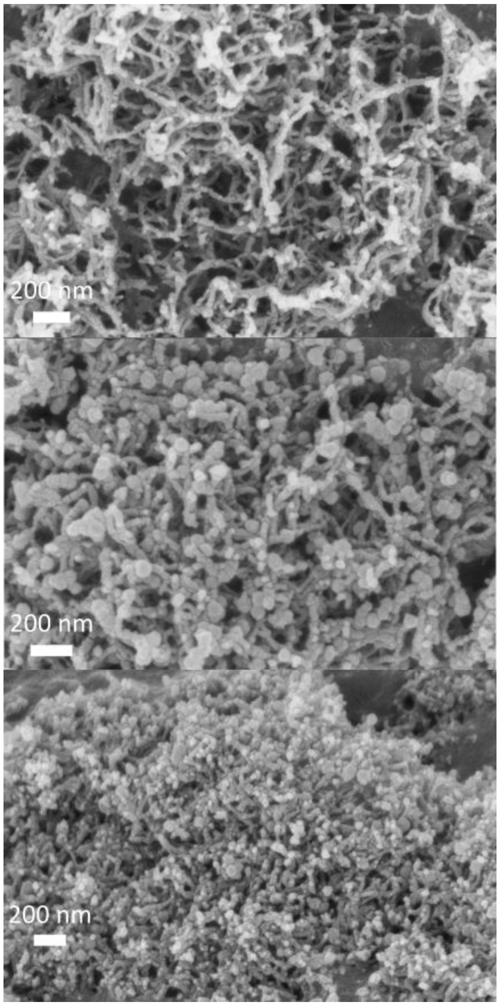

S16: FE-SEM images of NH_2-MOF-808/COF aerogels with different loadings. top: 25 %, middle: 50 %, bottom: 75 %.

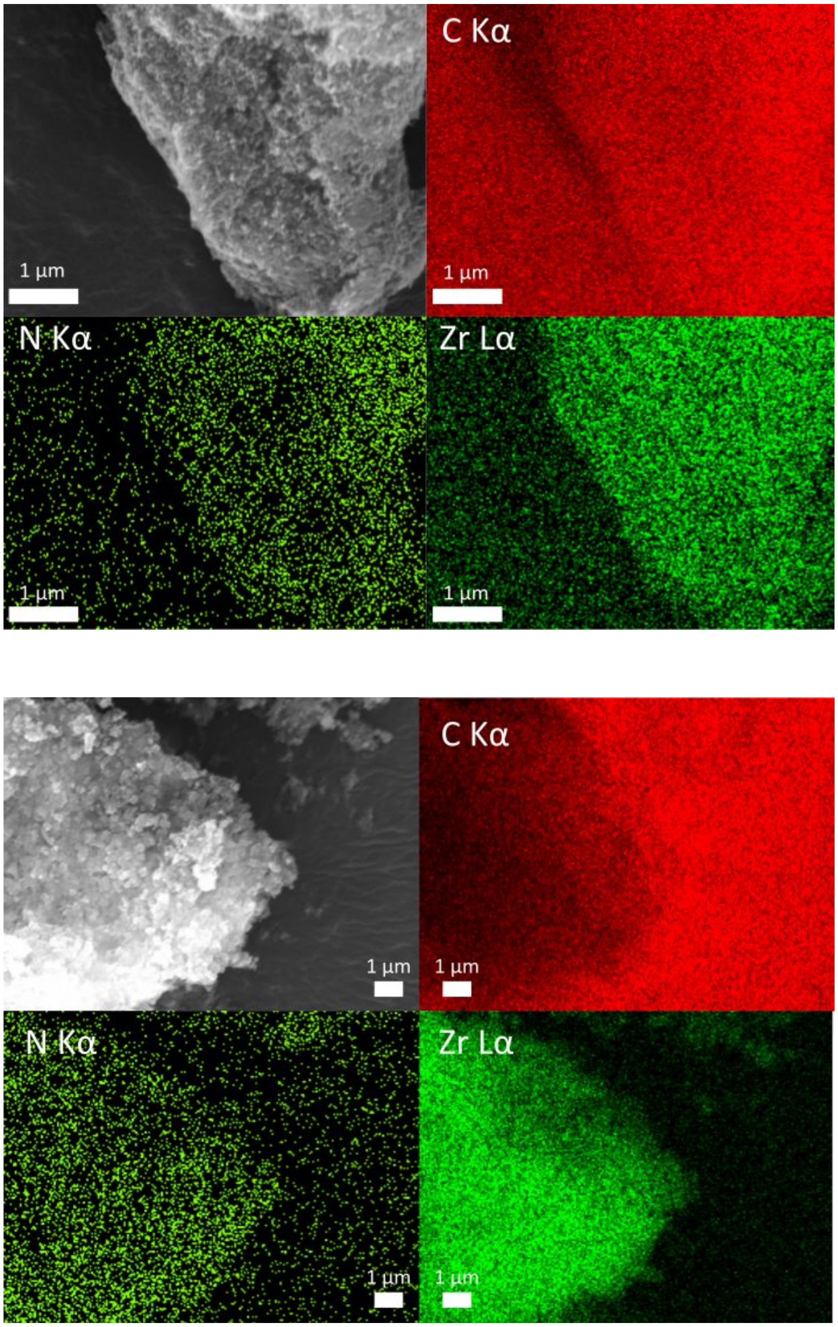

S17: EDX mapping of NH_2-MOF-808 (top) and NH_2-UiO-66/COF (bottom) aerogels.

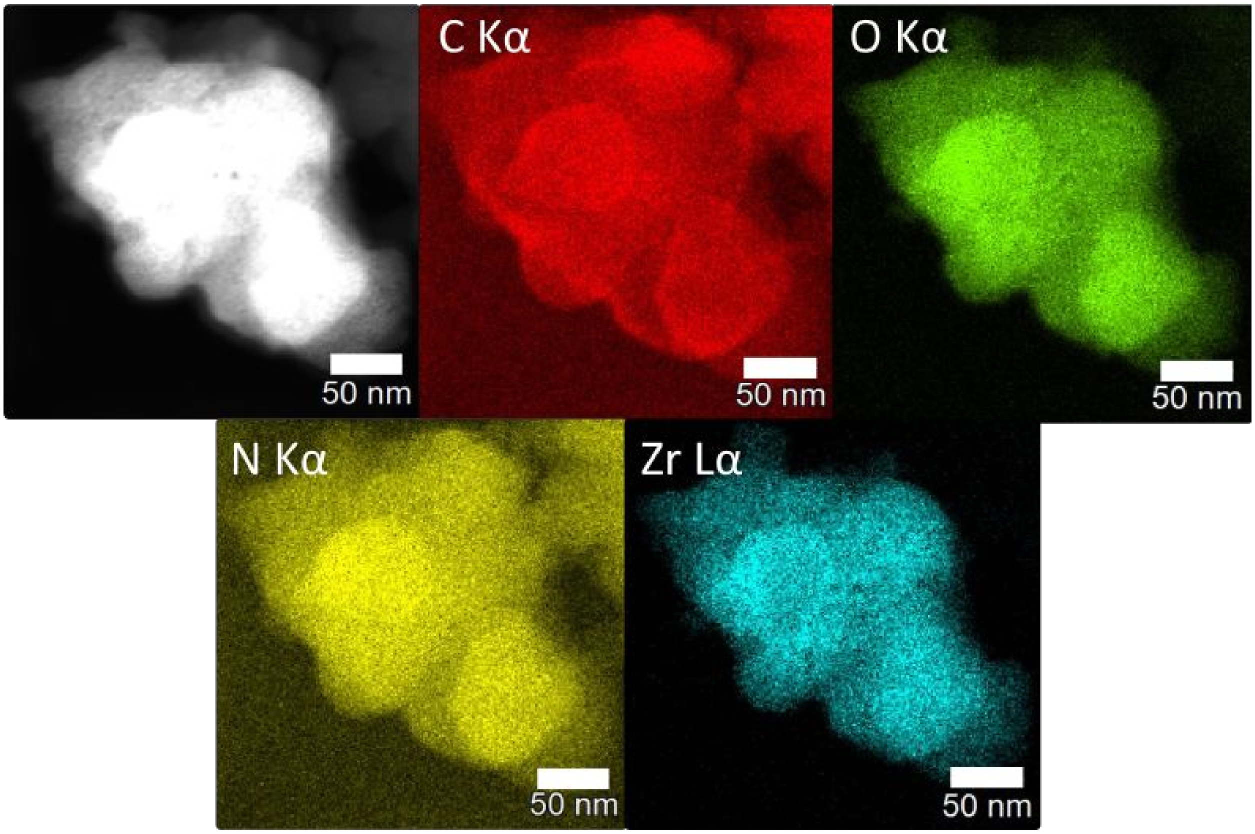

S18: STEM-EDX mapping of NH_2-MOF-808_{AB}/COF aerogel.

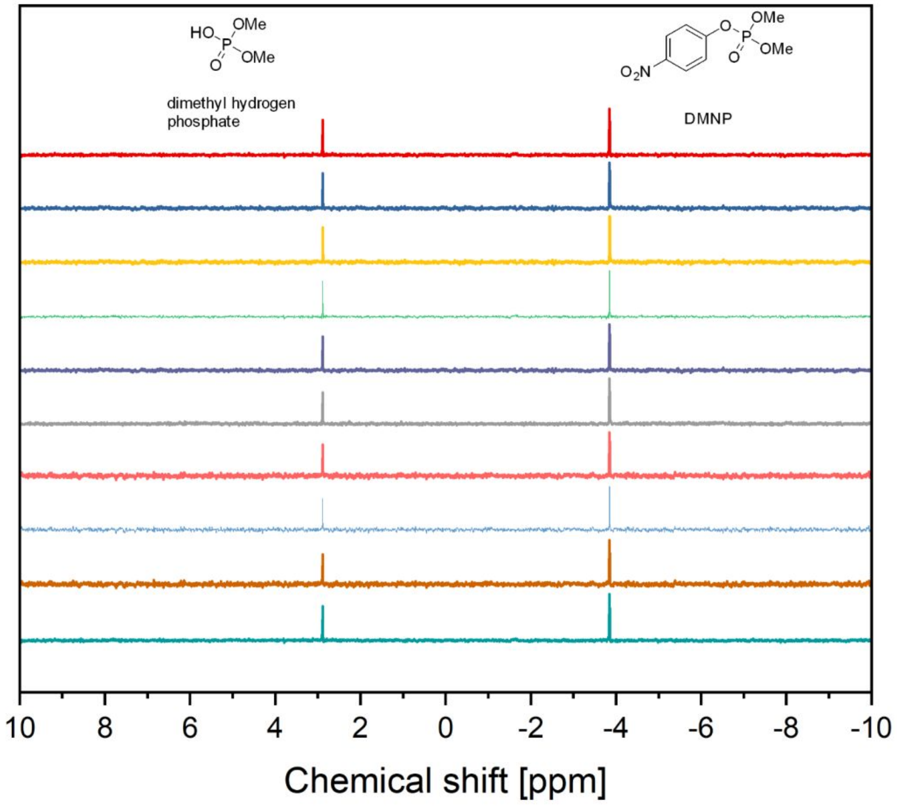

S19: DMNP Degradation using 25 mg NH_2-UiO-66/COF aerogel (Cycle 1 to 10 from bottom to top).

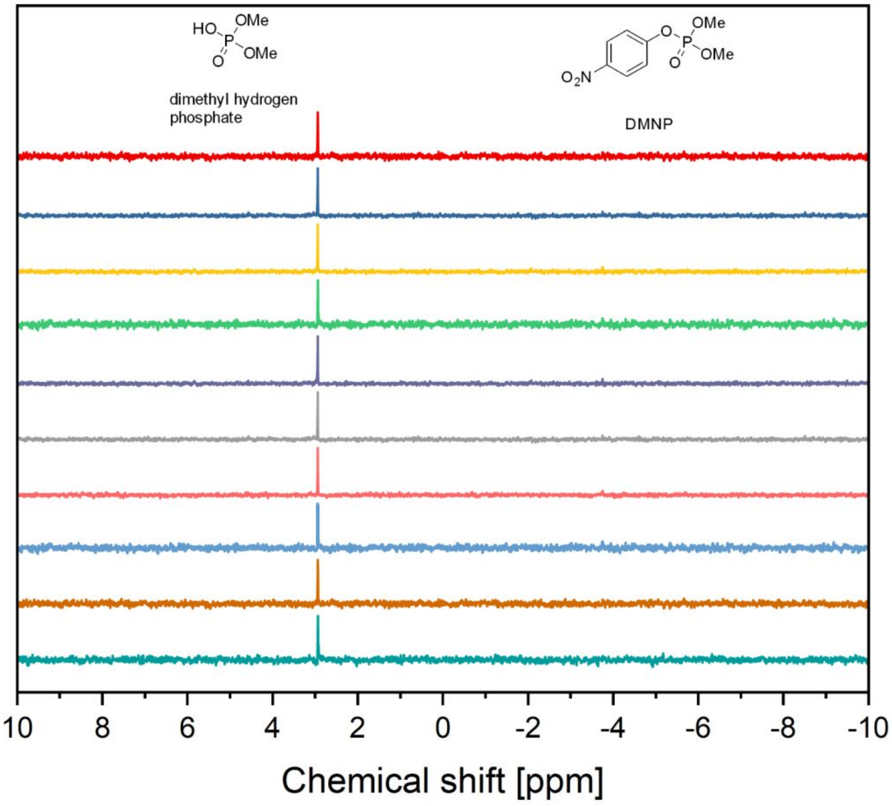

S20: DMNP Degradation using 25 mg NH_2-MOF-808/COF aerogel (Cycle 1 to 10 from bottom to top).

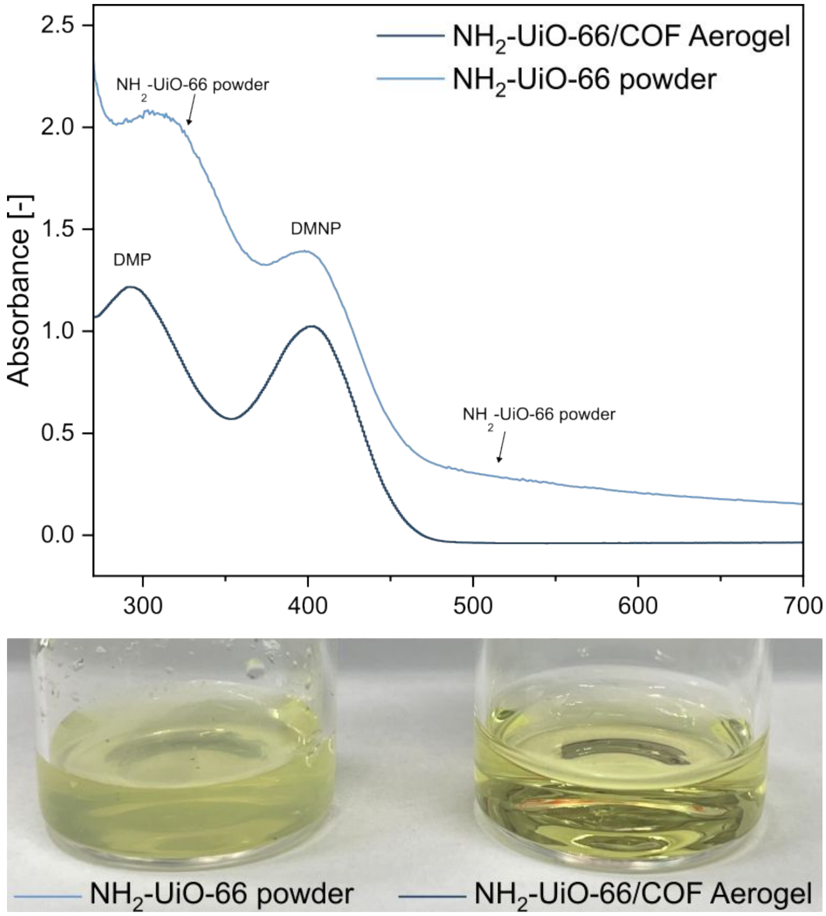

S21: Comparison of secondary pollution from DMNP filtration using MOF powder and MOF/COF aerogel.
